# A Conjugative IncI1 Plasmid Carrying *erm*(B) and *bla*_CTX-M-104_ That Mediates Resistance to Azithromycin and Cephalosporins

**DOI:** 10.1128/Spectrum.00286-21

**Published:** 2021-09-08

**Authors:** Xuemei Yang, Xiaoxuan Liu, Chen Yang, Edward Wai-Chi Chan, Rong Zhang, Sheng Chen

**Affiliations:** a Department of Infectious Diseases and Public Health, Jockey Club College of Veterinary Medicine and Life Sciences, City University of Hong Kong, Kowloon, Hong Kong; b State Key Laboratory of Chemical Biology and Drug Discovery, Department of Applied Biology and Chemical Technology, The Hong Kong Polytechnic University, Hung Hom, Hong Kong; c Department of Clinical Laboratory, Second Affiliated Hospital, Zhejiang University School of Medicine, Hangzhou, China; University of Texas Southwestern Medical Center

**Keywords:** *Klebsiella pneumoniae*, azithromycin resistance, colistin resistance, *erm*(B), *mcr-8*

## Abstract

In this study, an IncI1 plasmid encoding resistance to both cefotaxime and azithromycin was recovered from a clinical Klebsiella pneumoniae strain. The azithromycin resistance was confirmed to be mediated by the *erm*(B) gene. This plasmid could be readily conjugated to strains of Escherichia coli and Salmonella, promoting rapid dissemination of azithromycin- and ceftriaxone-resistance-encoding elements among Gram-negative bacterial pathogens. Transmission of this plasmid in Salmonella is of particular concern, since it could mediate expression of phenotypic resistance to azithromycin and ceftriaxone, which are the current choices for treatment of Salmonella infections. Our findings suggest a need to monitor the efficiency and pattern of transmission of this plasmid among key Gram-negative bacterial pathogens.

**IMPORTANCE** Since the approval by the FDA of azithromycin for treatment of Salmonella infections, efforts have been made to monitor the development of resistance to azithromycin in these organisms. In this study, we report an IncI1 plasmid from a clinical K. pneumoniae strain that encodes resistance to both cefotaxime and azithromycin. This plasmid could be readily conjugated to strains of Escherichia coli and Salmonella, promoting rapid dissemination of azithromycin- and ceftriaxone-resistance-encoding elements among Gram-negative bacterial pathogens. Furthermore, data from this study confirmed for the first time the role of the *erm*(B) gene in mediating resistance to azithromycin in various bacterial species, particularly Salmonella.

## INTRODUCTION

Azithromycin, a derivative of erythromycin, is a semisynthetic macrolide antibiotic (1, 2). It was generated by incorporating a nitrogen atom in the macrocyclic lactone ring of erythromycin, resulting in a stable 15-membered azalide (3). Since azithromycin exhibits reduced toxicity, improved pharmacokinetics, metabolic stability, and high tolerability, it has been used worldwide after being approved for clinical use in 1988 (3–5). Classic macrolides exhibit low levels of activity against *Enterobacteriaceae*, due to their poor membrane penetration potential (6). In contrast, azithromycin is membrane permeable and thus exhibits excellent potential for clinical treatment of infections caused by members of *Enterobacteriaceae*, such as Escherichia coli, *Shigella* spp., or Salmonella spp. (7, 8). Like other macrolides, azithromycin binds to the bacterial ribosome and inhibits mRNA translation, thus interfering with protein synthesis and preventing bacterial growth (6). Several mechanisms of resistance to macrolides have been described; one involves reduction in binding affinity of the drug via modification of either the bacterial ribosome or the drug molecule (9). A second mechanism is efflux of the drug from the bacterial cell via enhancement of efflux pump expression (10).

Klebsiella pneumoniae, which belongs to the *Enterobacteriaceae* family, is regarded as a reservoir of drug resistance genes due to its widespread presence among humans and in the environment (11). Although macrolides are not recommended for treatment of infections caused by K. pneumoniae, a variety of macrolide resistance genes in this species have been described (12). However, whether these genes confer cross-resistance to azithromycin remains unclear (10). In this report, we describe the identification of a conjugative plasmid harboring the erythromycin ribosome methylase gene, *erm*(B). The plasmid was recovered from a clinical K. pneumoniae strain that was confirmed to exhibit resistance to azithromycin. The *erm*(B) gene was demonstrated to mediate expression of phenotypic resistance to azithromycin in strains of various members of the *Enterobacteriaceae* family, particularly Salmonella, since azithromycin is an FDA-approved drug for treatment of clinical Salmonella infections. Widespread transmission of this kind of plasmid among clinical *Enterobacteriaceae* strains, or dissemination of clinical strains harboring such a plasmid, would result in significant reduction in the clinical value of azithromycin, thereby severely limiting the choices of effective antimicrobial agents for the treatment of life-threatening infections.

## RESULTS AND DISCUSSION

A strain suspected to be K. pneumoniae, namely, HK31, was recovered from a hospitalized patient in Hong Kong SAR in 2016 (Table 1). Antimicrobial susceptibility tests performed on strain HK31 showed that it was resistant to azithromycin, the β-lactam antibiotics ampicillin, aztreonam, cefotaxime, and ceftazidime, and the aminoglycoside antibiotics gentamicin and amikacin, as well as ciprofloxacin, chloramphenicol, and colistin, but remained susceptible to meropenem and tigecycline (Table 2).

**TABLE 1:** Strains and plasmids used in this study

Strain or plasmid	Relevant genotype^*a*^	Source
Strains		
E. coli		
DH5α	F^−^ Φ80*lac*ZΔM15 Δ(*lacZYA-argF*)*U169 recA1 endA1 hsdR17*(r_K_^−^ m_K_^+^) *phoA supE44 thi-1 gyrA96 relA1* λ^−^	Invitrogen
J53	Derivative of E. coli K-12; azide^r^	Laboratory stock
25922	Quality control strain	ATCC
K. pneumoniae		
HK31	Clinical strain; AZI^r^, *erm*(B)	This study
* S.* Typhimurium		
PY1 (14028s)	Derivative of CDC6516-60	ATCC
Plasmids		
pCR2.1 TOPO	Amp^r^, Kan^r^; pUC ori TA cloning vector, topoisomerase I	Invitrogen
pCR2.1/*erm*(B)	*erm*(B) in pCR2.1	This study

a azide^r^, sodium azide resistance; AZI^r^, azithromycin resistance; Amp^r^, ampicillin resistance; Kan^r^, Kan resistance.

**TABLE 2:** Phenotypic and genotypic characteristics of K. pneumoniae strain HK31 and its transconjugants

Strain	Species	MIC (μg/ml) for:^*a*^													
		AZI	CTX	CAZ	CIP	CHL	ATM	AMP	AMK	GEN	MEM	CLS	TGC	*erm*(B)^*b*^	Conjugation efficiency
HK31	K. pneumoniae	128	>128	>128	128	64	>128	>128	>128	>128	<0.25	32	0.5	+	NA
J53	E. coli	2	<0.25	0.5	<0.25	2	<0.25	8	2	2	<0.25	<0.25	<0.25	−	NA
J53TC	E. coli	64	>128	2	<0.25	2	64	>128	2	2	<0.25	<0.25	<0.25	+	1.33E−06
PY1	*S.* Typhimurium	4	<0.25	8	<0.25	4	<0.25	8	2	2	<0.25	1	0.5	−	NA
PY1TC	*S.* Typhimurium	64	>128	32	<0.25	4	128	>128	2	2	<0.25	1	0.5	+	3.5E−07
25922	E. coli	2	<0.25	0.5	<0.25	4	<0.25	8	2	4	<0.25	0.5	0.5	NA	NA

a All tests were performed in duplicate, and each test included three biological replicates. AZI, azithromycin; CTX, cefotaxime; CAZ, ceftazidime; CIP, ciprofloxacin; CHL, chloramphenicol; ATM, aztreonam; AMP, ampicillin; AMK, amikacin; GEN, gentamicin; MEM, meropenem; CLS, colistin; TGC, tigecycline.

b NA, not available.

Strain HK31 was then subjected to whole-genome sequencing using both the Illumina NextSeq 500 platform and the long-read Oxford Nanopore Technologies MinION platform. The genome size of strain HK31 was found to be 5,798,010 bp, including a 5.13-Mb chromosome and three plasmids with sizes of 204,580 bp, 115,662 bp, and 98,579 bp. Strain HK31 was found to belong to sequence type 273-1 locus variant (ST273-1LV [gapA-infB-mdh-pgi-phoE-rpoB-tonB: 3-4-6-1-7*-4-4]) based on multilocus sequence typing (MLST) and the KL14 serotype based on capsular typing. BLAST searches against the resistance gene database showed that this strain harbored multiple resistance genes, including the quinolone resistance genes *qnrB4* and *oqxAB*, the β-lactam resistance genes *bla*_CTX-M-15_, *bla*_CTX-M-104_, *bla*_SHV-11_, *bla*_SHV-12_, and *bla*_DHA-1_, the aminoglycoside resistance genes *armA*, *ant(2″)-Ia*, and *aph(3′)-Ia*, the macrolide resistance genes *mph*(E), *msr*(E), and *erm*(B), the fosfomycin resistance gene *fosA*, the sulfonamide resistance gene *sul1*, and the colistin resistance gene *mcr-8*. The *bla*_CTX-M-15_ and *bla*_CTX-M-104_ variants were both 876 bp in length and showed 100% coverage and 100% identity to the reference sequences. Except for the *bla*_SHV-11_, *fosA*, and *oqxAB* genes, the resistance genes were found to be located in plasmids. The *fosA* gene was flanked by the transcriptional regulator gene *lysR* upstream and the homocysteine *S*-methyltransferase gene downstream. No mobile genetic elements were found surrounding the *fosA* gene. However, the *fosA* gene from the chromosome of K. pneumoniae can be captured by E. coli and disseminated through mobile elements (13).

**FIG 1:**
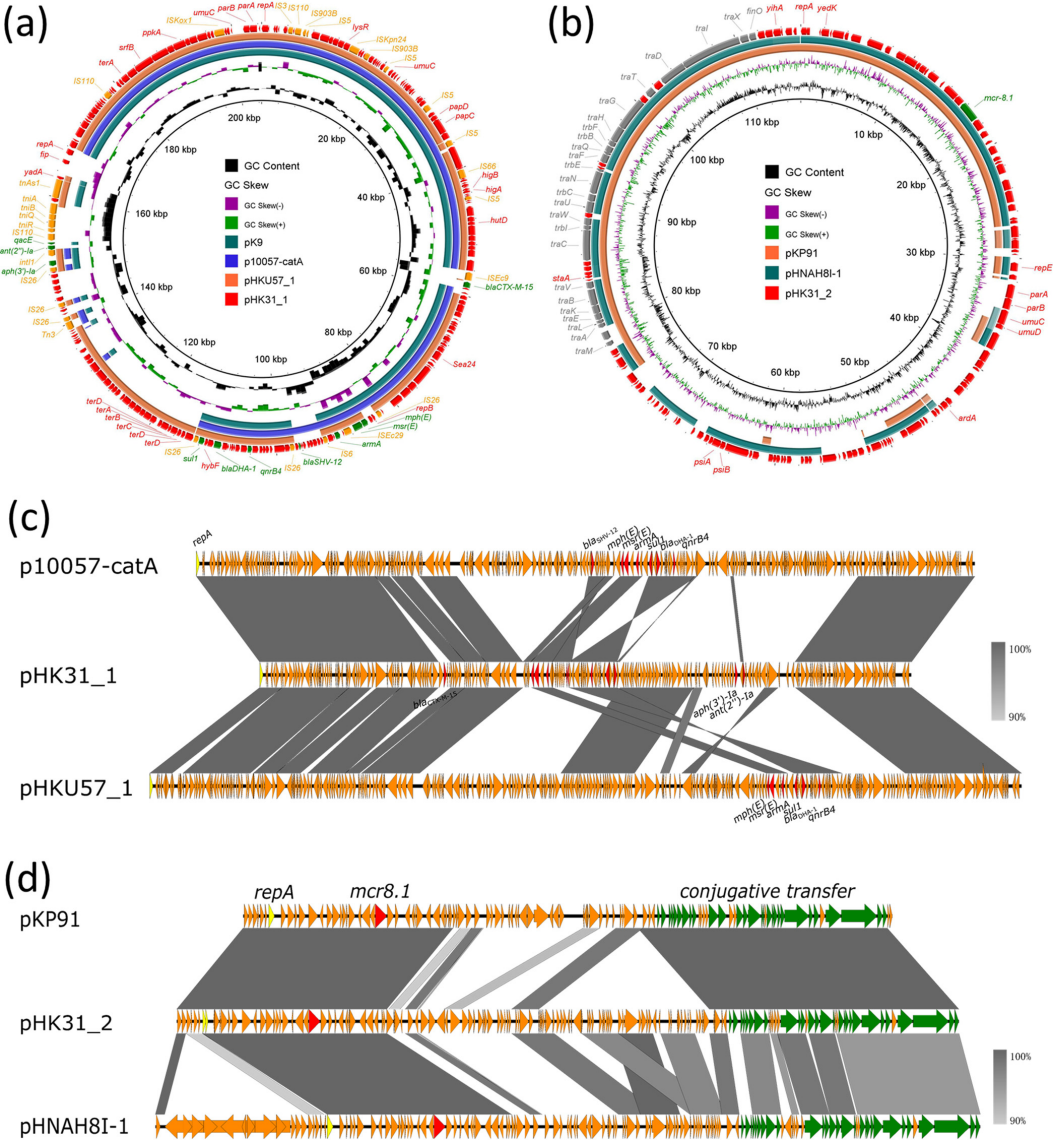
Alignment of plasmids pHK31_1 and pHK31_2 with structurally similar plasmids by BRIG and Easyfig. (a) Plasmid pHK31_1 exhibits the highest degree of similarity (77% coverage and 99.88% identity) to plasmid p10057-catA (GenBank accession number MN423364.1). (b) Plasmid pHK31_2 exhibits the highest degree of similarity to pKP91 (GenBank accession number MG736312.1) (72% coverage and 99.72% identity) and plasmid pHNAH8I-1 (GenBank accession number MK347425.1) (84% coverage and 99.12% identity). (c) Alignment of plasmid pHK31_1 with plasmids p10057-catA and pHKU57_1 using Easyfig. (d) Alignment of plasmid pHK31_2 with plasmids pKP91 and pHNAH8I-1 using Easyfig.

The *qnrB4*, *bla*_CTX-M-15_, *bla*_SHV-12_, *bla*_DHA-1_, *armA*, *ant(2″)-Ia*, *aph(3′)-Ia*, *mph*(E), *msr*(E), and *sul1* genes were found to be located in the 204,580-bp plasmid, which was designated pHK31_1 (Fig. 1a). This plasmid was an IncFIB_K_ plasmid, possessed 239 coding sequences with a GC content of 50.3%, and exhibited the highest degree of sequence similarity (77% coverage and 99.88% identity) to a 244,456-bp IncFIB_K_ plasmid known as p10057-catA (GenBank accession number MN423364.1). The 273,789-bp IncFIB_K_/IncHI1B plasmid pHKU57_1 (GenBank accession number CP063216.1) and the 324,283-bp plasmid pK9 (GenBank accession number CP049891.1), both recovered from K. pneumoniae strains, also harbored similar backbones (Fig. 1a and c). The *mcr-8* gene was found to be located in the 115,662-bp plasmid, which was designated pHK31_2 (Fig. 1b). Plasmid pHK31_2 was an IncFIA(HI1)/IncFII_K_ plasmid, possessing 150 coding sequences with a GC content of 51.6%. It exhibited the highest degree of sequence similarity to a 95,983-bp IncFIA(HI1)/IncFII_K_ plasmid known as pKP91 (GenBank accession number MG736312.1) (72% coverage and 99.72% identity) (14) and a 121,961-bp IncFIA(HI1) plasmid known as pHNAH8I-1 (GenBank accession number MK347425.1) (84% coverage and 99.12% identity) (15), both recovered from K. pneumoniae strains (Fig. 1b and d). Although plasmid pHK31_2 carried *tra* genes, this plasmid seemed not to be conjugative, based on our conjugation assay results after many trials.

**FIG 2:**
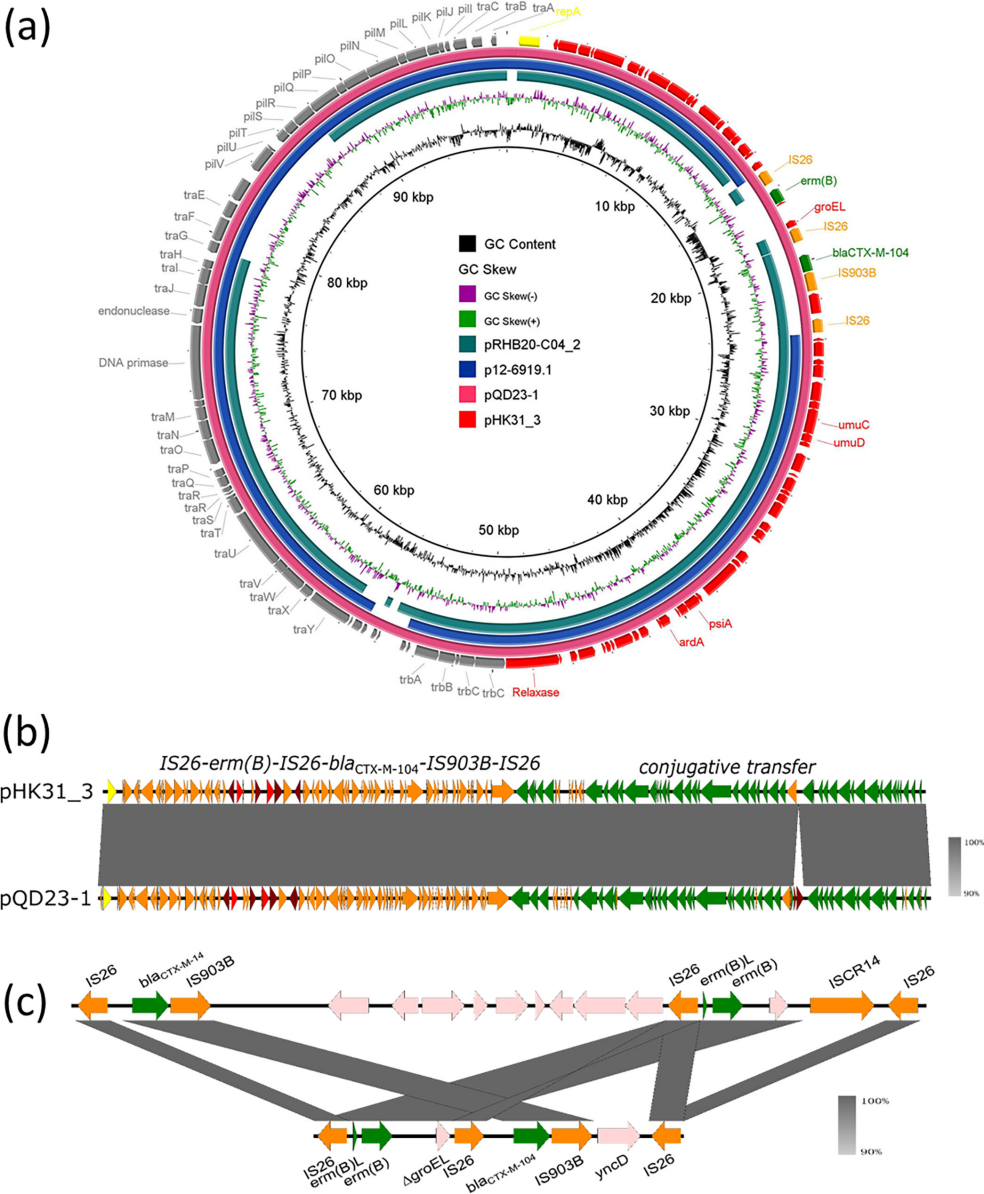
Alignment of plasmid pHK31_3 with structurally similar plasmids by BRIG. (a) Plasmid pHK31_3 exhibits the highest level of similarity (100% coverage and 99.75% identity) to plasmid pQD23-1 (GenBank accession number MN548042.1). Plasmid p12-6919.1 (GenBank accession number CP039604.1) from Salmonella enterica subsp. *enterica* serovar strain PNCS014868 and plasmid pRHB20-C04_2 (GenBank accession number CP057639.1) from E. coli strain RHB20-C04 harbored similar plasmid backbones but lacked the mosaic resistance region. (b) Alignment of plasmid pHK31_3 with plasmid pQD23-1 using Easyfig. (c) The *erm*(B) gene in plasmid pHK31_3 is located in the truncated Tn*6295* element within the plasmid p2246-CTXM.

The *bla*_CTX-M-104_ and *erm*(B) genes were found to be located in the 98,579-bp plasmid, which was designated pHK31_3 (Fig. 2). This plasmid was an IncI1-type plasmid, possessing 119 protein-coding genes with a GC content of 49.8%. It exhibited the highest degree of sequence similarity (100% coverage and 99.75% identity) to plasmid pQD23-1 (GenBank accession number MN548042.1), which is a 99,802-bp IncI1 plasmid recovered from K. pneumoniae strain QD23. The 90,972-bp IncI1 plasmid p12-6919.1 (GenBank accession number CP039604.1) from Salmonella enterica subsp. *enterica* serovar strain PNCS014868 and the 109,773-bp IncI1 plasmid pRHB20-C04_2 (GenBank accession number CP057639.1) from E. coli strain RHB20-C04 harbored similar backbones but lacked the mosaic resistance region (Fig. 2a and b). The *erm*(B) gene was found to be located in a truncated Tn*6295* element harbored by plasmid p2246-CTXM, which was recovered from a Shigella boydii strain (16). Plasmid p2246-CTXM also harbored a *bla*_CTX-M-14_ gene flanked by IS*903B*, indicating that the fragments bearing *erm*(B) and *bla*_CTX-M-104_ in plasmid pHK31_3 might have originated from this kind of plasmid (Fig. 2c).

The *erm*(B) gene, which belongs to the *erm* family, encodes the enzyme erythromycin ribosome methylase, which catalyzes dimethylation of the nucleotide A2058 in 23S rRNA to prevent drug binding (17). To test its effect on azithromycin, the *erm*(B) gene and its leading peptide-encoding sequence *erm*(B)*L* in plasmid pHK31_3, were cloned and introduced into E. coli strain DH5α and *S*. Typhimurium strain PY1. The MICs of strains DH5α and PY1 that had acquired the *erm*(B) gene increased to more than 128 μg/ml (Table 3). The results indicated that *erm*(B) also encoded resistance to azithromycin.

**TABLE 3:** MICs of strains that had acquired the *erm*(B) gene via transformation

Strain	Azithromycin MIC (μg/ml)
DH5α	2
DH5α/*erm*(B)	>128
PY1	4
PY1/*erm*(B)	>128

**FIG 3:**
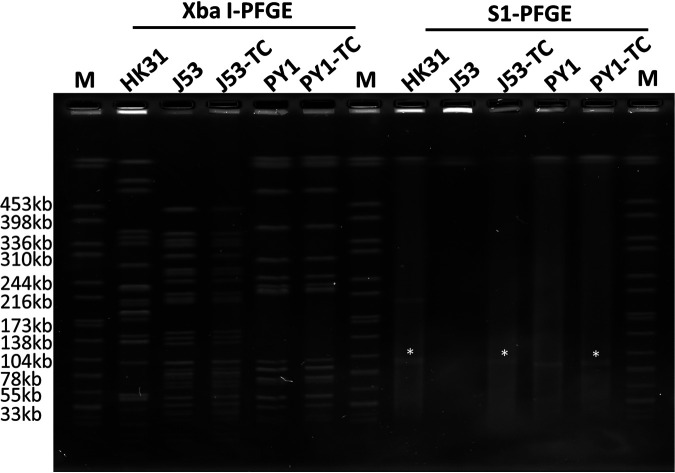
XbaI PFGE and S1 PFGE analyses of strain HK31, recipient strain E. coli J53, *S.* Typhimurium PY1, and their corresponding transconjugants J53TC and PY1TC. Asterisks denote the conjugative plasmid pHK31_3. XbaI PFGE and S1 PFGE were repeated twice for all strains to confirm the consistency of the XbaI PFGE and S1 PFGE profiles.

Because pHK31_3 was shown to carry the *tra* gene cluster of the IncI plasmid, which encodes plasmid conjugation functions, a conjugation experiment was then performed to test its potential to be transferred to strains of E. coli and Salmonella species, which are common pathogens that belong to the *Enterobacteriaceae* family. The results showed that pHK31_3 could be directly conjugated from strain HK31 to E. coli strain J53, with an efficiency of 1.33E−06 (Table 2 and Fig. 3). The transconjugant obtained was found to exhibit XbaI pulsed-field gel electrophoresis (PFGE) profiles that were identical to those of the recipient strain, but there was evidence of acquisition of the 100-kb plasmid according to the S1 nuclease PFGE profiles (Fig. 3). A transconjugant strain of J53 was then treated as the donor to conjugate this plasmid to the Salmonella enterica subsp. *enterica* serovar Typhimurium PY1 recipient strain. The results showed that plasmid pHK31_3 could be directly conjugated from strain J53 to strain PY1 with an efficiency of 3.5E−07 (Table 2 and Fig. 3). These transconjugants were also confirmed to have acquired the ability to express phenotypic resistance to azithromycin and ceftriaxone (Table 2). The ability to conjugate such a plasmid to Salmonella spp. highlighted the clinical significance of this conjugative plasmid encoding both azithromycin and cephalosporin resistance, since these two types of antibiotics are current choices for treatment of clinical Salmonella infections. Since the approval by the FDA of azithromycin for treatment of Salmonella infections, efforts have been made to monitor the development of resistance to azithromycin in these organisms. Azithromycin resistance has been increasingly reported in Salmonella strains, while the exact mechanisms remain unclear. The *mphA* gene has been shown to be associated with azithromycin resistance in Salmonella strains, while a large proportion of *mphA*-positive Salmonella strains in our collection are susceptible to azithromycin (unpublished data, Chen KC and Chen S), which contradicts the role of *mphA* as an azithromycin resistance gene in Salmonella. Data from this study confirmed for the first time the role of the *erm*(B) gene in mediating resistance to azithromycin in various bacterial species, particularly Salmonella.

## CONCLUSION

In this study, we characterized a conjugative plasmid that harbored the *erm*(B) gene from a clinical K. pneumoniae strain and confirmed the role of this gene in mediating azithromycin resistance in various bacterial species. This plasmid was also able to conjugate to strains of E. coli and Salmonella and encode phenotypic azithromycin and ceftriaxone resistance. Surveillance and close monitoring of the transmission of this plasmid in Gram-negative bacteria, particularly Salmonella, should be implemented.

## MATERIALS AND METHODS

### Bacterial strains, plasmids, and growth conditions.

Strains and plasmids used in this work are listed in Table 1. K. pneumoniae strain HK31 was isolated from a clinical patient in a hospital located in Hong Kong SAR and was identified by the Vitek 2 system (bioMérieux, France), with confirmation by matrix-assisted laser desorption ionization–time of flight mass spectrometry (MALDI-TOF MS) (Bruker, Germany). E. coli strains 25922 and J53 and *S.* Typhimurium strain PY1 were from our laboratory stocks. Strains were grown in Luria-Bertani (LB) broth at 37°C. Kanamycin (Kan) was added at a concentration of 50 μg/ml where appropriate.

### Cloning of the *erm*(B) gene.

The *erm*(B) gene was amplified using the primers ermB-F (AGAAGGAGGGATTCGTCATG) and ermB-R (TCTTGCTAGTCTAGGGACCT). Briefly, fragments of 200 bp upstream and downstream of the *erm*(B) gene were amplified, ligated into the pCR2.1 TOPO vector (Invitrogen), and introduced into E. coli DH5α by heat shock at 42°C. The resulting plasmid recoverable from the transformants was verified by sequencing and electroporated into *S.* Typhimurium PY1.

### Conjugation assay.

To evaluate the transferability of the azithromycin-resistance-encoding plasmid, sodium azide (NaN_3_)-resistant E. coli strain J53 was used as the recipient, as described previously (18). Transconjugants were screened by using eosin-methylene blue (EMB) agar plates containing 8 μg/ml azithromycin and 100 μg/ml NaN_3_. The presence of the *erm*(B) gene as a marker gene in the plasmid harbored by the transconjugants was determined by PCR. The MIC profiles of the transconjugants were determined to differ from those of the donor. XbaI PFGE and S1 nuclease PFGE were performed to confirm the transfer of this plasmid. Successful transconjugants of E. coli strain J53 were then treated as the donor and *S.* Typhimurium strain PY1 was used as the recipient to further determine the transferability of this plasmid. XLT4 agar plates containing 8 μg/ml azithromycin were used to select transconjugants. To calculate the conjugation efficiency, the culture obtained after conjugation was diluted and spread on plates containing only 100 μg/ml NaN_3_, with strain J53 being used as the recipient, as well as plates without antibiotics, with strain PY1 being used as the recipient. The conjugation efficiency was calculated as the number of transconjugant cells divided by the number of recipient cells.

### Antibiotic susceptibility tests.

Antimicrobial susceptibility of the test strains was determined by using the microdilution method according to the guidelines provided by the Clinical and Laboratory Standards Institute (19). Antimicrobial agents including azithromycin, cefotaxime, ceftazidime, ciprofloxacin, chloramphenicol, aztreonam, ampicillin, gentamicin, amikacin, meropenem, colistin, and tigecycline were tested. All tests were performed in duplicate; each test included three biological replicates per strain.

### DNA sequencing and bioinformatics.

Genomic DNA from strain HK31 was extracted using the genomic purification kit for bacteria (Qiagen, Germany) according to the manufacturer’s instructions. The extracted DNA was then subjected to library preparation with the NEBNext Ultra II DNA library preparation kit for Illumina (New England Biolabs, USA) and sequenced via the 150-bp paired-end NextSeq 500 platform (Illumina, San Diego, CA). Genomic DNA was also subjected to sequencing with the long-read MinION platform, following the manufacturer’s guide (Oxford Nanopore Technologies, Oxford, United Kingdom). Both short and long reads were *de novo* hybrid assembled using Unicycler v0.4.8 (20). Assembled genome sequences were annotated with RAST v2.0 (21). MLST was performed by Kleborate software based on genetic variation in seven housekeeping genes (22). Capsular typing of the assembled sequences was performed using Kaptive (23). The BLAST command lines, with 80% coverage and identity cutoff values, were used to map genome sequences against antibiotic resistance genes and plasmid replicons. The resistance gene and plasmid replicon databases were obtained from the Center for Genomic Epidemiology (http://www.genomicepidemiology.org). Alignment of plasmid sequences with similar structures was generated by BLAST Ring Image Generator (BRIG) v0.95.22 (24) and Easyfig_win_2.1 (25).

### Data availability.

All sequencing data have been deposited in GenBank under BioProject accession number PRJNA725664. GenBank accession numbers CP073906, CP073907, CP073908, and CP073909 have been assigned to sequences of the strain HK31 chromosome, plasmid pHK31_1, pHK31_2, and pHK31_p3, respectively.
